# Do Not Miss the Tumor: A Novel Presentation of Osteosarcoma

**DOI:** 10.1155/2021/5531238

**Published:** 2021-05-13

**Authors:** Michael DePalma, Sachin Gupta, Jie Nguyen, Divya Talwar, Alexandre Arkader, Lawrence Wells

**Affiliations:** ^1^Children's Hospital of Philadelphia, Philadelphia, PA, USA; ^2^Perelman School of Medicine, University of Pennsylvania, Philadelphia, PA, USA

## Abstract

Antalgic gait is a common clinical presentation among pediatric patients and can have many different etiologies, with rare life-threatening etiologies including primary bone malignancies. Osteosarcoma is the most common primary malignancy of bone in pediatric and adolescent patients. The incidence rate of osteosarcoma has been reported as high as 5 to 7 per million among patients 19 years old or younger with males slightly more affected than females and African-Americans more than other racial groups. This report describes the case of a five-year-old African-American female who presented with an antalgic gait secondary to osteosarcoma in the left distal femur and follows her through treatment. In this case, the age is atypical as the peak incidence for osteosarcoma is around 16 years of age and is postulated to coincide with growth spurts. Osteosarcoma can have a range of presentations making it difficult to diagnose, which can cause delays in treatment and potential poor patient outcomes. Due to this, such a diagnosis must be included in the differential for patients presenting with antalgic gait. Because primary-care physicians and pediatricians may be the first medical providers to encounter patients with osteosarcoma, it is imperative that such clinicians are familiar with the signs and symptoms associated with osteosarcomas in order to reduce the risk of metastasis and disease progression and prevent treatment delays. Additionally, we believe these clinicians should have a low threshold to refer patients to orthopedists or oncologic specialists in the cases of persistent pain or inconsistencies with history, physical exam, and diagnostic studies. Finally, direct communication and discussion between radiologists and referring clinicians helps decrease delays in diagnosing of osteosarcoma and other life-threatening conditions.

## 1. Introduction

Antalgic gait is a common clinical presentation among pediatric patients and can have many different etiologies. The most common causes include trauma, infection, inflammatory or congenital malformations, and metabolic imbalances [1]. Less common causes include life-threatening etiologies such as primary bone malignancies (i.e., osteosarcoma and Ewing sarcoma) [1]. Osteosarcoma is the most common primary malignancy of bone in pediatric and adolescent patients [2–4]. The incidence rate of osteosarcoma has been reported as high as 5 to 7 per million among patients 19 years old or younger with males slightly more affected than females and African-Americans more than other racial groups [4].

Patients with osteosarcoma can have a variety of clinical presentations, which can complicate and delay both diagnosis and appropriate treatment. Often, patients will present with localized pain after a recent injury, which may fluctuate but is usually worse at night [2]. Patients may also present with or without a palpable mass at the site of injury with warmth and tenderness to palpation [2]. Osteosarcomas most commonly arise at the level of the distal femur or proximal tibia, so pain at this site may exacerbate with weight bearing, often manifesting as an antalgic gait in clinic [2]. Systemic symptoms may include weight loss, fever, fatigue, and malaise; however, patients without these symptoms should not be ruled out for osteosarcoma [2]. Typical radiographic findings will show mixed osteolytic and osteoblastic areas with aggressive periosteal involvement and a wide zone of transition between normal and abnormal bone [3].

The 5 year survival rate has been reported as high as 61.6% in patients 19 years old or younger with neoadjuvant chemotherapy and surgical intervention utilized for local control. The 5 year survival rate decreases significantly in patients with distant metastases, most notably the lungs and other bones [5]. Therefore, early detection, particularly by primary-care physicians and general orthopedists, is key for these patients [2, 4]. Delays in diagnosis may have devastating effects on patient outcomes, which supports the need for clinicians to include rare conditions such as osteosarcomas in their systematic approach to patients presenting with antalgic gait [4]. This report describes a case of a five-year-old African-American female who presented with an antalgic gait secondary to osteosarcoma in the left distal femur and follows her through treatment. In this case, the age is atypical as the peak incidence is around 16 years of age and postulated to coincide with growth spurts [1–3].

## 2. Case

**Figure 1:**
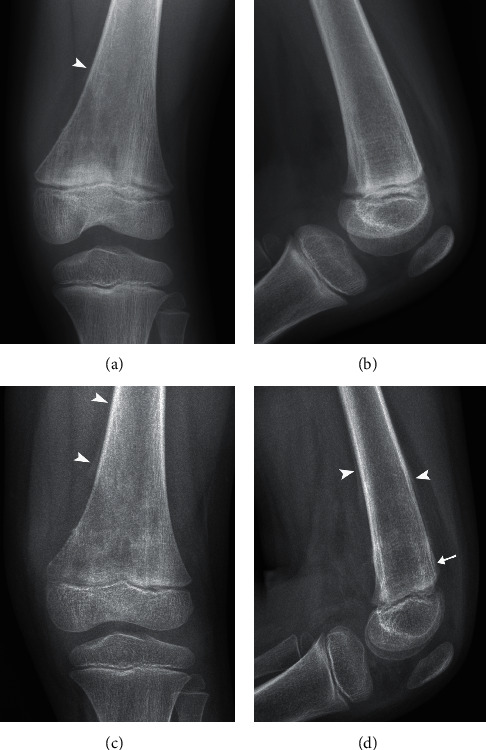
Anteroposterior and lateral knee radiographs at presentation (a, b) and 15 days later (c, d) show interval progressive medullary-based eccentric lytic change with a wide zone of transition, aggressive (Codman's triangle) periosteal reaction (arrowheads), and subtle cortical deformity (arrow).

This patient, a five-year-old African-American female, fell at school and developed severe left knee pain. Three days after falling, the patient's mother contacted her primary-care physician where she reported her daughter's severe left knee pain that prevented her from walking and had worsened since the initial injury. At this point, the patient's left knee was also warm and erythematous. She and her daughter were referred to her local emergency department where radiographs of the patient's left knee were conducted and were read as unremarkable with no signs of trauma (Figure 1). The patient was, therefore, diagnosed with a left knee sprain and was treated conservatively with pain control (ice and NSAIDs) and NWB without further intervention.

Eleven days later, the patient was seen by her primary-care physician for her persistent limp and intermittent pain, although her swelling had resolved. Anteroposterior view of the pelvis and bilateral frog lateral radiographs were ordered to rule out any hip pathologies, which could also cause an antalgic gait. All films were read by an attending radiologist and were deemed unremarkable. Relying on these reads, the primary-care physician recommended the patient to continue supportive care (NSAIDs and ice for pain, nonweight bearing for left lower extremity) and to refer to an orthopedist if symptoms persisted.

**Figure 2:**
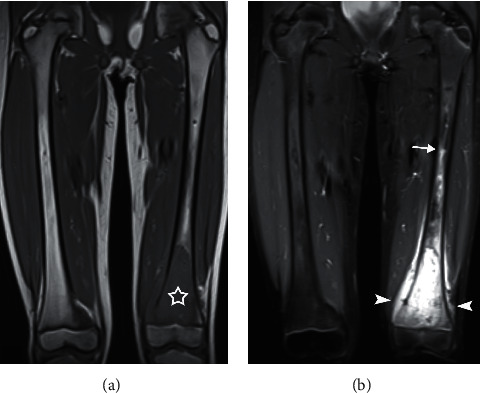
Magnetic resonance imaging (MRI) of the thighs. Coronal T1-weighted (a) and fluid-sensitive (b) large-field-of-view images confirm a marrow replacement process centered within the left distal femoral metaphysis (star) with extraosseous extension (arrowheads) and proximal marked peritumoral bone marrow edema-like signal (arrow).

Despite these treatments, the patient's pain and limp persisted, and the patient then sought a referral to a general pediatric orthopedist for further work-up. Although the child's pain had slightly improved, her limp remained. She described her left knee pain as worse at night but responsive to acetaminophen and ice. She had never felt anything like this before prior to her initial fall. Both the patient and her mother denied fever, weight loss, fatigue, malaise, or recent infection. On physical exam, her left knee did not have a palpable mass, muscle atrophy, or edema, but was tender to palpation. Given concern for the persistent pain and antalgic gait, repeat radiographs of the left knee were also ordered by the orthopedist and showed aggressive poorly defined lytic lesion centered within the left distal femur metadiaphysis extending up proximally 6 cm superiorly from the metaphysis with associated interrupted superior periosteal involvement (Figure 1). A radiologist reviewed these findings and agreed with the orthopedist that based on these radiographic findings, there was concern for a primary bone malignancy. Arthrocentesis of the left knee was performed to rule out a possible infectious etiology, which was negative. Acute-phase reactants including c-reactive protein, sedimentation rate, and leukocyte count were all within normal limits. Magnetic resonance imaging (MRI) and needle biopsy were ordered to establish diagnosis. Contrast-enhanced MRI examination showed a enhancing mass centered in the distal femoral metaphysis approximately 7 cm in craniocaudal dimension with aggressive periosteal involvement (Figure 2). Needle biopsy of the left distal femur was performed at the site of the lesion, and pathology demonstrated cores of tumor tissue consisting of pleomorphic tumor cells with frequent mitoses, foci of necrosis, regions of chondroblastic differentiation, and rare small foci of malignant osteoid production, consistent with the diagnosis of osteosarcoma. Additional imaging, including chest computerized tomography (CT) and whole body Positron Emission Tomography (PET), was obtained for tumor staging and demonstrated no evidence of distant metastases.

The patient was then referred to an orthopedic surgeon to discuss treatment options including oncologic and operative interventions. Given the concern for pathological fracture and possible subsequent contamination of healthy tissue from this tumor, the patient was placed in a knee immobilizer to limit weight bearing (NWB LLE with walker). The patient and her parents were referred to an oncologist to determine a chemotherapy plan. A typical treatment plan of osteosarcoma involves surgical resection and six cycles of chemotherapy (two cycles preoperatively as neoadjuvant therapy and four cycles postoperatively) with high-dose methotrexate, doxorubicin/dexrazoxane, and cisplatin.

Operative treatments were discussed between the family and the attending orthopaedic surgeon in great detail including potential risks and benefits, and the family chose to proceed with Van Nes Rotationplasty. Radical resection of the left distal femur 17 cm from the joint line with femur/tibia osteoplasty with internal fixation and Van Nes Rotationplasty of the left lower extremity and left sciatic neuroplasty was performed. The procedure lasted around eight hours long without complications. Estimated blood loss was 100 mL or less. The patient was transferred to the PICU for monitoring postoperatively. There were no wound complications postoperatively, and the patient received trimethoprim-sulfamethoxazole and cefepime for antibiotic prophylaxis. Nine days later, the patient was discharged with referrals to physical therapy and the appropriative postoperative wound care instructions.

**Figure 3:**
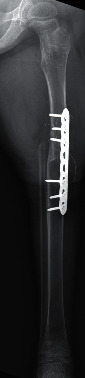
Postoperative anteroposterior radiograph shows resection of the distal femur with Van Nes rotationplasty.

The patient was treated with chemotherapy for five months postoperatively and responded well. Since finishing chemotherapy treatments, the patient has continued with physical therapy and has been fitted for a lower extremity prosthetic. Postoperative radiographs have demonstrated appropriate healing of the rotationplasty (Figure 3). Six-month postoperative Positron Emission Tomography (PET) showed no evidence of local or distant metastatic disease. There was minimal uptake in the left lower extremity at the location of the rotationplasty. The procedure with the appropriate adjuvant chemotherapy was deemed successful.

## 3. Discussion

Osteosarcoma is the most common primary malignant bone tumor in pediatric and adolescent patients, but can have a range of presentations making it difficult to diagnose, which can cause delays in treatment and potential poor patient outcomes [2, 4].

This case highlights the ambiguity of symptoms that can cloud such a diagnosis. This patient presented with an antalgic gait without a palpable mass. However, with further investigation, it was discovered the patient did have “red-flag” symptoms including pain at night and worsening pain inconsistent with the initial injury. Although radiographs were ordered, they were incorrectly read by both the attending radiologist and primary-care physician. This was an unfortunate occurrence but often occurs when the radiology read is “handed-off” to the patient and physicians neglect to look at the actual images [6]. Therefore, although radiographs are the sensible first step in diagnosis, in this instance, they were futile because they did not improve efficiency in diagnosis and treatment. Fortunately, this did not affect the overall outcome of the patient. It is well known that early detection and initiation of treatment is vital for patients with osteosarcoma because of the significant risk of metastases and associated poorer prognosis with increased tumor volume [5]. Primary-care physicians encountering patients with an antalgic gait should have a low threshold for not only taking repeat imaging but also examining images themselves. These physicians should also have a low threshold for referral to an orthopaedic surgeon should a patient's presenting complaint or history be inconsistent with their exam or diagnostic studies [5, 7]. It is imperative that such clinicians are familiar with the signs and symptoms associated with osteosarcomas in order to reduce the risk of metastasis and disease progression and prevent treatment delays [1, 4, 5, 7–9].

Radiologists also play a vital role in the care team for patients with osteosarcoma. In addition to evaluating the character and extent of the disease, radiologists are essential in the timely communication of diagnostic information to other members of the osteosarcoma care team [10]. Larson et al. have demonstrated that modern information systems allow ample time for radiologists to directly contact clinicians via telephone or other means of communication [6]. The electronic sharing of diagnostic results can improve patient outcomes, particularly in malignant diseases such as osteosarcoma where timely treatment is crucial for patient survival [6].

This case highlights the systematic diagnostic approach needed for patients presenting with antalgic gait. Due to the high risk of malignancy and poorer prognosis associated with metastasis, osteosarcoma must be diagnosed and treated without delay [5]. Primary-care physicians and pediatricians play a crucial role in this early detection and, therefore, must be familiar with the common features of osteosarcoma. In addition, such a diagnosis must be included in the differential for patients presenting with antalgic gait [9]. Similarly, we believe primary-care physicians and pediatricians should have a low threshold to refer patients to orthopedists or oncologic specialists in the cases of persistent pain or inconsistencies with history, physical exam, and diagnostic studies [9, 10]. Finally, direct communication and discussion between radiologists and referring clinicians helps decrease delays in diagnosing osteosarcoma and other life-threatening conditions [6, 10].

## Data Availability

The clinical data used to support the findings of this study are included within the article.
